# Seedling-stage drought responses of two endemic pear and oak species inform climate-adaptive management in Hyrcanian forests

**DOI:** 10.3389/fpls.2025.1715389

**Published:** 2026-02-25

**Authors:** Yadollah Davoudi, Masoud Tabari, Seyed Ehsan Sadati, Martin Karl-Friedrich Bader

**Affiliations:** 1 Department of Forest Science and Engineering, Faculty of Natural Resources, Tarbiat Modares University, Tehran, Iran; 2 Research Division of Natural Resources, Mazandaran Agricultural and Natural Resources Research and Education Center, AREEO, Sari, Iran; 3 Department of System Earth Science, Faculty of Science and Engineering, Brightlands Campus Greenport Venlo, Venlo, Netherlands

**Keywords:** drought stress, water deficit, forest tree seedlings, electrolyte leakage, malondialdehyde, proline

## Abstract

**Introduction:**

The diverse Hyrcanian relic forests in the Caspian region are increasingly threatened by more frequent and severe climate change-related droughts. This study investigated morphophysiological and biochemical responses to drought in two endemic tree species of the Hyrcanian forests, *Pyrus boisseriana* Buhse and *Quercus atropatena* Schwarz.

**Methods:**

In a 120-day greenhouse experiment, seedlings of each species were exposed to two different irrigation treatments (FC100 and FC40, representing the percentage of field capacity).

**Results:**

Under FC100 conditions, all morphological characteristics of *Q. atropatena* were significantly greater compared to those of *P. boisseriana*. FC40-exposed *P. boisseriana* and *Q. atropatena* seedlings exhibited statistically significant declines in photosynthesis (-55, -49.6%), transpiration (-11.8, -21.7%), intercellular CO_2_ concentration (*C*_i_) (-54.8, -8.3%), mesophyll conductance (*g*_m_) (-58.5, -49.5%) and RWC (only *Q. atropatena*: -22%), respectively, and an increase in leaf temperature. Likewise, FC40-treated *P. boisseriana* and *Q. atropatena* seedlings displayed an increase in proline (+390.8, +46.5%), and a decline in carotenoids (-19.9, -14.5%), respectively. Drought stress had smaller impacts on radial and height growth, photosynthesis, *C*_i_, *g*_m_, EL and MDA, in *Q. atropatena* compared to *P. boisseriana*, indicating greater drought tolerance in the former.

**Discussion:**

These findings imply a prioritization of *Q. atropatena* in forest management and conservation planning in arid and semi-arid Hyrcanian forests, especially given future climate scenarios. Our results also offer valuable insights for nursery managers facing water scarcity and for stakeholders involved in afforestation and reforestation initiatives aimed at cultivating these two species. Since tree species selection is essential to maintaining forest sustainability, our research adds to the ongoing discussion about the drought tolerance of tree species during the critical seedling stage, particularly in the context of climate change.

## 1 Introduction

Severe droughts are expected to increase in frequency, magnitude, and spatial extent in the coming decades posing significant risks to the supply of essential resources such as water, food, and energy (Alencar and Paton, 2025; Teutschbein et al., 2025). The frequency of drought events has grown over the past century, particularly in the last fifty years (Cai et al., 2021). Recent projections from the World Climate Research Program indicate that global warming will impact the hydrological cycle, leading to heightened risks and intensified drought conditions in certain regions by the century’s end, including the Hyrcanian forests, which have increasingly experienced dry spells and reduced precipitation in recent years. Concurrently, soil drying is expected to become increasingly widespread and intense with rising temperatures (Cook et al., 2020). The distribution of plant species will be profoundly affected by such environmental changes. However, the degree of these consequences will vary based on the ability of each species to withstand water scarcity (Zia et al., 2021). Approximately 50% of the Earth’s surface is covered by forests in which keystone tree species play a pivotal role in ecosystem structure and functioning by providing essential habitats, food sources, and symbiotic relationships for a variety of microorganisms, fungi, animals, and other plant species (Aitken et al., 2008). Comprehending how trees cope with drought and its consequences on tree health and functioning is essential for the effective management of forest resources, the identification of suitable species and origins in reforested regions, and the conservation of forest ecosystems (Robakowski et al., 2021).

Plants cannot migrate from environments with unfavorable conditions like animals, and are therefore susceptible to extreme weather conditions (Seleiman et al., 2021). Water deficit is considered one of the most important environmental stresses with negative effects on plant growth, development and intracellular processes (Trenberth et al., 2014). Drought represents a significant constraint on plant growth, as the process of cell expansion – driven by the hydrostatic pressure within the newly forming cells – requires adequate water availability in the cambium (Peters et al., 2021). Water scarcity impedes essential physiological processes such as photosynthesis (*A*), respiration, and the movement of stomata, ultimately influencing overall plant growth and metabolic functions (Yang et al., 2021). Drought-induced impairment of photosystem II (PSII) and photosystem I (PSI) activity restricts photosynthetic electron transport, leading to the accumulation of excess energy in the reaction centers and consequently enhancing the formation of reactive oxygen species (ROS) and oxidative damage (Zhang et al., 2020). Photosynthesis (*A*) is indicative of the biomass productivity per unit leaf area, thus serving as a dependable metric for assessing the overall production capacity of plants under otherwise non-limiting conditions. The closure of stomata leads to a reduction in CO_2_ absorption, resulting in a diminished (*A*). In seedlings that lack large carbon reserves, prolonged stomatal closure may lead to carbon limitation, which not only reduces biomass productivity but may also hasten drought-related mortality (Reddy et al., 2019; Liu et al., 2022). This phenomenon is strongly influenced by the intensity and duration of drought events, as well as the developmental stage of the plant (Tardieu et al., 2018). Overall, drought stress may result in a decrease in survival (Engelbrecht and Kursar, 2003), growth (Xiaoqin et al., 2009), leaf area (Wang et al., 2024b), and plant biomass (Abbaspour et al., 2012). Depending on the level of drought adaptation, plant water relations deteriorate more or less rapidly (Fang and Xiong, 2015; Shivakrishna et al., 2018; Farooq et al., 2012), and, at the same time, oxidative damage indicated by oxidative stress markers increases (e.g., MDA, hydrogen peroxide, and elektrolyte leakage (EL) (Anjum et al., 2011), which is often accompanied by a rise in antioxidants and osmoregulants (Sachan et al., 2020).

*Pyrus boissieriana* Buhse (Rosaceae) is the second most widely distributed wild pear species across Iran. It primarily thrives in the Hyrcanian Forest region (Zakavi et al., 2016), which is distinguished by a range of ecological conditions, including annual precipitation levels between 213 and 2045 mm and elevations from 12 m below sea level to 2400 m a.s.l., contributing to its extensive biodiversity (Heidari et al., 2019). Various researchers have classified wild pear species as xerophytic woody plants due to their comparatively low reliance on soil moisture (Boucek, 1954; Zakavi et al., 2016). Previous studies revealed that *P. boissiriana* specimens originating from semi-arid environments exhibit greater tolerance to drought stress compared to specimens from semi-humid populations (Zarafshar et al., 2014a) and these more drought hardy individuals may thus serve as valuable rootstock resources for more drought-sensitive commercial wild pear scions in agricultural settings (Zakavi et al., 2016). It has been reported that wild pear trees under drought stress experience a decrease in photosynthetic pigment concentration (Sattarian et al., 2015), RWC and water potential, and an increase in EL (Zarafshar et al., 2018). *Quercus atropatena* O. Schwarz & Hess is a tree of the Fagaceae family native to the Hyrcanian forests of Iran, which is of great ecological importance. Although reports on the characteristics of this species are few, studies have shown that the oak genus is generally tolerant to water deficit stress (Khosravi et al., 2022; Hernandez and Park, 2022). However, despite the wealth of oak-related drought studies, there are no reports on the physiological and biochemical responses of *Q. atropatena* to drought conditions.

In recent decades, increasing population growth and water demand have highlighted the importance of water resources management in arid and semi-arid regions, including Iran, marked by low rainfall and irregular distribution (Tabari and Talaee, 2011). To thrive in these harsh conditions, plants require powerful resistance and/or tolerance mechanisms (Zerga, 2015). In arid and semi-arid regions, expanding forests and increasing afforestation is hindered by limited water resources, which makes the selection of drought-resistant species crucial. Therefore, screening for drought tolerance traits and assessing water requirements is one of the most effective approaches to water management and ensuring success in seedling planting projects in these regions. There is a lack of information on the drought sensitivity of *P. boissieriana* and *Q. atropatena*, which are potential candidates for reforestation initiatives. The aim of this study was to assess the morphological, physiological, and biochemical responses of *P. boissieriana* and *Q. atropatena* seedlings to drought stress to inform forest managers and other stakeholders about their potential in future forest planning and decision-making. In this study, we seek to answer the following questions: 1) How do the morphological, physiological and biochemical responses differ between these two species under drought stress conditions? 2) What promise do *P. boissieriana* and *Q. atropatena* hold as drought-tolerant species for future forest regeneration in this increasingly dry region?

## 2 Materials and methods

### 2.1 Experimental design

In this study, two-year-old seedlings of *Pyrus boissieriana* and *Quercus atropatena* grown in 5-liter containers were evaluated under controlled greenhouse conditions at the Lajim Nursery in northern Iran. The Lajim Forest Nursery, located in the central district of Savadkuh County (53°07’ E, 36°16’ N) at an elevation of approximately 750 m above sea level, approximately 30 km northeast of Pol-e Sefid and 35 km southeast of Sari. The site is situated within the Hyrcanian forest region of northern Iran and is characterized by a temperate climate with cool winters and dry summers. Seeds of *Quercus atropatena* originated from naturally occurring mother trees distributed along the 1300–1500 m elevational belt in the Kelenga area of Neka County, whereas seeds of *Pyrus boissieriana* were collected from wild populations located at 900–1000 m elevation in the Margab region of Sari County. For this purpose, 192 healthy seedlings (96 seedlings from each species) with approximately the same diameter (8 ± 2 mm) and height (40 ± 3 cm) were selected. This investigation was performed using a factorial experimental approach, structured as a randomized complete block design, with three replications. The seedlings were planted in plastic pots (5 liters) filled with loamy–sandy soil. The soil texture (particle size distribution), bulk density, and moisture content were measured in the soil laboratory of the Mazandaran Research Center (**Table 1**). Based on these measurements, the soil water retention curve, which describes the relationship between soil water potential and moisture content, was plotted using the method of Saxton et al. (1986). Accordingly, the field capacity of the soil was determined. By calculating the difference between field capacity and permanent wilting point, as well as their percentage, the reference weight for soil moisture control was established. This reference weight included the dry weight (DW) of soil, seedling biomass, pot weight, and the reference weight at the wilting point. Once the reference weight was determined, irrigation intervals were scheduled accordingly. To minimize surface evaporation, the lateral sides of the pots were covered with aluminum foil. Therefore, during each irrigation treatment (FC100, 100% field capacity; FC40, 40% field capacity), the pots were weighed and then irrigated with a graduated cylinder (beaker) to the amount equal to the difference from the reference weight (Sadati et al., 2019).

**Table 1:** Physical and chemical properties of the potting soil.

Soil texture	Sand (%)	Silt (%)	Clay (%)	pH	Electrical conductivity (dS/m)	Potassium (ppm)	Phosphorus (ppm)	Total nitrogen (%)	Organic matter (%)
Loamy–sandy	52	36	12	7.0	1.2	150	20	0.25	3.0

### 2.2 Morphological characteristics

Morphological characteristics, including root diameter (RD) and seedling height (SH), were measured at the beginning and end of the experiment. RD was measured using a digital caliper (with an accuracy of one millimeter) and height was measured using a graduated ruler (with an accuracy of one cm). From the difference between diameter and also height at the end and beginning of the experiment, diameter growth and height growth were obtained, respectively. Then, three seedlings were randomly chosen from each treatment, removed from the soil and after carefully washing off rhizosphere soil, the root length was measured using a ruler (Parad et al., 2016) and the root volume was measured from the change in water volume in the graduated cylinder (Azizi et al., 2020). Leaf, root, and stem samples were oven-dried at 70 °C for 48 hours. Subsequently, biomass was determined using a digital scale with a precision of 0.0001 g. The total biomass for the seedlings was calculated by combining the dry weight (DW) of their root, leaves, and shoot. A leaf area meter (Model LI-3000, Li-Cor, Lincoln, NE, USA) was employed to assess the leaf area of six fully developed leaves from the upper section of shoot in each seedling. The specific leaf area (SLA) was determined by calculating the ratio of the leaf area to the DW of the leaves (Zarafshar et al., 2014b).

### 2.3 Physiological characteristics

We assessed leaf net photosynthetic rate (*A*), transpiration (E), stomatal conductance (g_s_), leaf temperature, and intercellular CO_2_ concentration (*C*_i_). Measurements were taken following treatments under natural conditions of temperature, light, and relative humidity between 9:30 and 11:00 AM, utilizing a portable gas exchange measurement device (Model LCpro+, ADC BioScientific Ltd., Hertfordshire, UK). For this analysis, 3–6 fully expanded, mature leaves were chosen from each replicate, located at the upper sections of the seedlings (Ghanbary et al., 2020). Relative mesophyll conductance (g_m_-rel) was estimated as the ratio *A/C^i^* following Fischer et al. (1998), who used Amax/Ci as an apparent mesophyll conductance index. In order to determine the RWC, three healthy and fully developed leaves were taken from the top of each seedling. The initial fresh weight (FW) of the leaves was documented. Subsequently, the leaves were submerged in distilled water in a dark environment for 24 hours to facilitate water absorption and swelling (SW). After this period, the fully hydrated leaves were weighed again followed by oven-drying at 70 °C for 48 hours, and subsequent DW determination. The RWC was then calculated using Equation 1 (Yang et al., 2007).

(1) RWC=[(FW−DW)/(SW−DW)]×100

### 2.4 Measurement of biochemical characteristics

The assessment of EL was conducted following the protocol outlined by Campos et al. (2003). Small leaf segments, each measuring 0.25 cm², were excised from 100 mg of fresh leaf tissue and placed into 50 mL falcon tubes filled with 15 mL of double distilled water. The tubes were then subjected to a boiling water bath at 80 °C for 12 hours. After 2 hours, the initial electrical conductivity (EC1) was measured using an electrical conductivity meter. The samples were then transferred to a non-ventilated oven set at 120 °C for 120 minutes. After cooling to 25 °C, the final electrical conductivity (EC2) was recorded. The EL was calculated using the following Equation 2:

(2) EL=[EC1/EC2]×100

To assess the pigment amount, 0.1 g of frozen leaf discs was mixed with 0.1 g of calcium carbonate and 4 mL of 80% acetone. This mixture was then extracted in the dark at −80 °C. The resulting solution was transferred to a separate test tube and centrifuged at 4000 rpm for 10 minutes at 4 °C. After the supernatant was separated, absorbance measurements were recorded spectrophotometrically at wavelengths of 470, 645, and 663 nm (PG Instruments T60, Wibtoft, Leicestershire, UK). The concentrations of chlorophyll a, chlorophyll b, and carotenoids were calculated using Equations 3, 4 and 5 (Arnon, 1949).

Here, V = the volume of acetone used (mL) and W = the weight of the sample leaf tissue (g). Pigment concentrations are given in mg g^−1^ fresh weight (FW). The total chlorophyll content was calculated by summing up the amounts of chlorophyll a and chlorophyll b.

(3) $$Chl\;a\;concentration=12.7\left(A663\right)-2.69\left(A645\right)\times \frac{V}{W\times 1000}$$

(4) $$Chl\;b\;concentration=22.9\left(A645\right)-4.68\left(A663\right)\times \frac{V}{W\times 1000}$$

(5) $$Carotenoids\;concentration=\left(A470\right)-14.4\left(A663\right)-63.08\;\left(A645\right)\times \frac{V}{W\times 1000}$$

Proline was measured following Bates et al. (1973). For this purpose, 0.5 g of frozen leaves from each species and treatment level were extracted using 5 mL of 3% sulfosalicylic acid. The resulting extract was then centrifuged for 15 minutes at 6000 rpm and 4 °C. Following this, 2 mL of the supernatant was combined with 2 mL of ninhydrin reagent, 30 mL of glacial acetic acid, and 2 mL of acetic acid, and the mixture was incubated in a hot water bath at 95 °C for one hour. After cooling on ice, 4 mL of toluene was introduced to the samples, which were then vortexed for 15 minutes after being kept in the dark for 20 minutes. The absorption of the pink upper phase containing toluene and proline was measured at 520 nm using a spectrophotometer (PG Instruments T60, Wibtoft, Leicestershire, UK), and the concentration of proline was determined by referencing a standard curve prepared with concentrations of 50, 40, 30, 20, 10, 5, 2.5, and 0 μM of pure proline.

In order to assess the MDA concentration, 0.2 g of frozen leaf discs from each species were mixed with 4 mL of trichloroacetic acid (TCA) buffer solution and subsequently subjected to centrifugation at 1500 rpm for 15 minutes. Following this step, 2 mL of the supernatant was extracted, and 2.5 mL of a 5% thiobarbituric acid (TBA) solution was added to it. The mixture was incubated in a hot water bath at 95 °C for 50 minutes, followed by centrifugation at 10, 000 rpm for 10 minutes. The mixture was cooled in ice water for 10 minutes, and the absorbance was measured spectrophotometrically at 532 nm (PG Instruments T60, Wibtoft, Leicestershire, UK). The concentration of MDA was determined using an extinction coefficient of 155 mm^−1^ cm^−1^ (Heath and Packer, 1968).

### 2.5 Statistical analysis

All statistical analyses were performed with SAS version 9.1 statistical software (SAS Institute Inc. 2023). Initially, the data’s homogeneity and normality were evaluated using the Levene test and the Kolmogorov-Smirnov test, respectively. A two-way ANOVA was then conducted to examine the interaction effects between species type and water deficit stress. We used graphical model validation tools to check the underlying assumptions. A plot of the standardized residuals vs. fitted values indicated no gross violation of the variance homogeneity assumption and a quantile-quantile plot of the residuals showed no strong deviation from normality. Duncan’s multiple range test was used as a *post hoc* procedure to compare the group means. Structural equation modeling (SEM) was conducted using Amos 24.0 software to assess the impact of species type and water deficit stress on various morphological, physiological and biochemical parameters. This approach was employed as it facilitates the exploration and comprehension of intricate connections among different morphological, physiological, and biochemical factors in relation to drought stress, offering insights into both the direct and indirect impacts of drought on the two examined species. In this research, a general structural equation model was utilized, linking observed variables to latent variables. The model was assessed through path coefficients to measure the strength and direction of these connections, where positive coefficients signify direct and beneficial relationships, while negative coefficients denote inverse relationships. Furthermore, Gephi 0.10 software served as a network modeling tool for constructing, visualizing and analyzing the network model.

## 3 Results

### 3.1 Morphological characteristics

**Figure 1:**
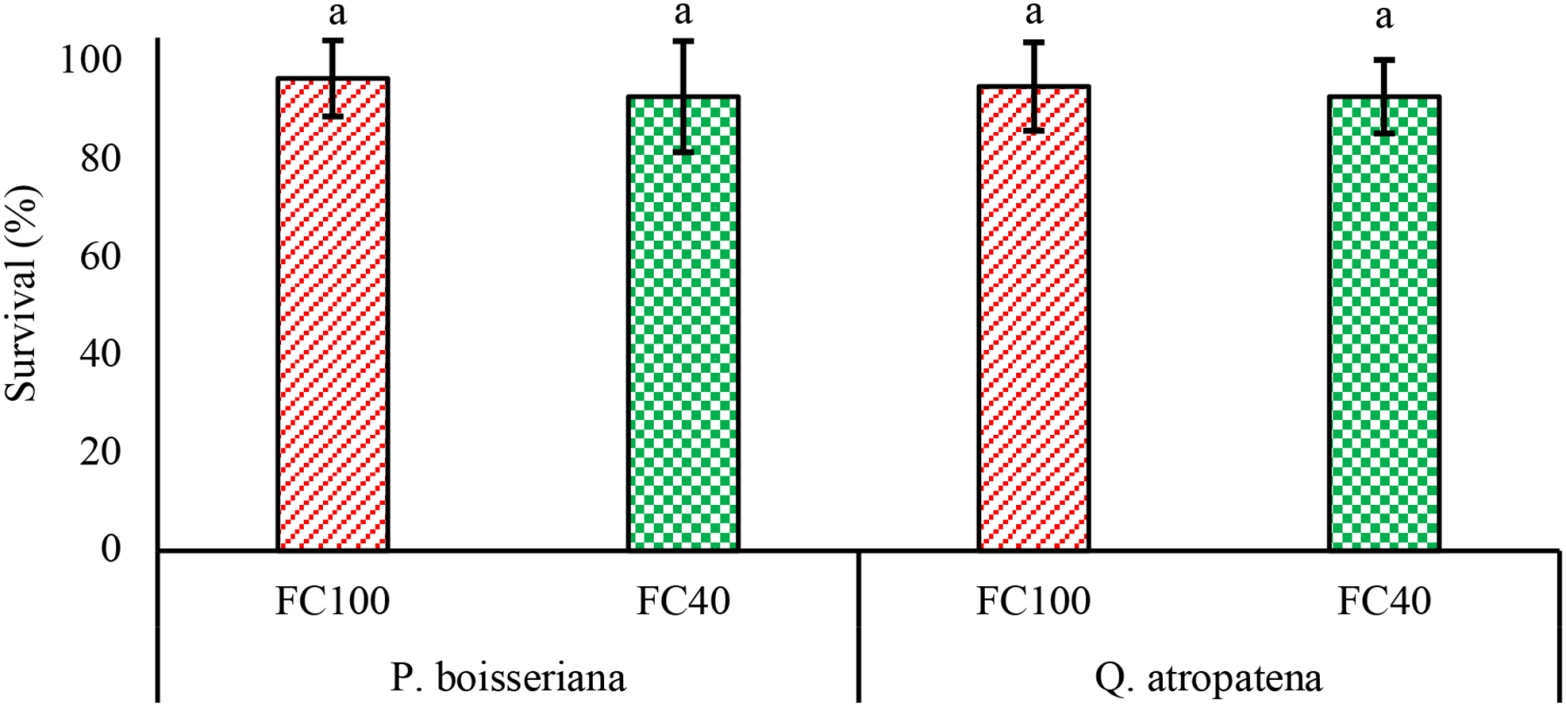
Means of seedling survival of *Quercus atropatena* Schwarz. and *Pyrus boisseriana* Buhse. seedlings under water deficit. Different lowercase letters indicate statistically significant differences in means among the 4 groups derived from a *post hoc* analysis (α = 0.05).

**Figure 2:**
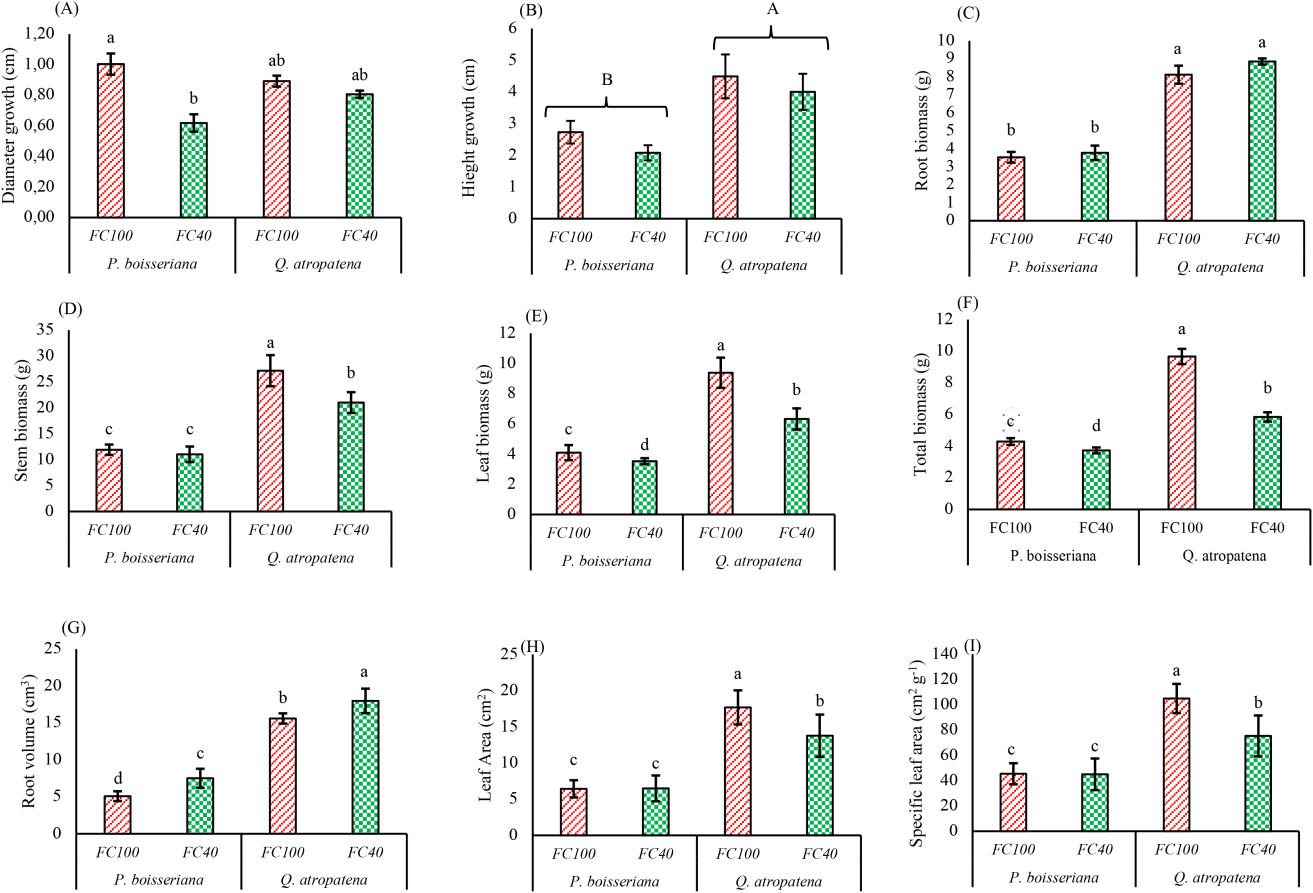
Means of root volume **(A)**, diameter growth **(B)**, height growth **(C)**, root biomass **(D)**, stem biomass **(E)**, leaf biomass **(F)**, total biomass **(G)**, leaf area **(H)** and specific leaf area **(I)** of *Quercus atropatena* and *Pyrus boisseriana* seedlings under water deficit. Different lowercase letters indicate statistically significant differences between means among the 4 groups, derived from a *post hoc* analysis (α = 0.05). Different uppercase letters indicate statistically significant differences between two species, regardless of the irrigation treatment (i.e. when the species × water deficit interaction was not significant).

The results of the two-way analysis of variance (**Table 2**) demonstrated that the interaction of species and water deficit had a significant effect *(P< 0.05*) on the morphological characteristics of *Q. atropatena* and *P. boisseriana* seedlings (except for survival, height growth and root biomass). The impact of species on the morphological traits of seedlings (except for survival and diameter growth) was significant (*P<0.01*). Also, water deficit stress had a considerable influence (*P<0.05*) on the morphological traits (except for survival, height growth, diameter growth, and root biomass) (**Table 2**). At the FC100 and FC40 field capacities, the survival rates of wild pear seedlings were 97% and 93%, and for oak seedlings 95% and 93%, respectively (**Figure 1**). The greatest and smallest diameter growth (examined for 6 months) occurred in wild pear seedlings at FC100 and FC40 with averages of 10 mm and 6 mm, respectively. Under stress conditions (FC40), diameter growth experienced a decline of 38.4% in wild pear and 9.6% in oak when compared to well-watered seedlings (FC100) (**Figure 2A**). This means that under drought conditions, radial growth losses in oak were four times lower than in wild pear.

**Table 2:** Two-way analysis of variance of the effect of species type, water deficit and their interaction on morphological, physiological and biochemical properties of *P. boissieriana* and *Q. atropatena* seedlings.

Property	Species			Water deficit			Species × Water stress		
	*df*	MS	*F* value	*df*	MS	*F* value	*df*	MS	*F* value
Morphological									
Survival	1	8.333	0.10ns	1	102.083	1.24ns	3	8.333	0.10ns
Diameter growth	1	0.070	0.15ns	1	1.077	2.27ns	3	2.664	5.62*
Height growth	1	162.803	13.73**	1	0.316	0.03ns	3	15.526	1.31ns
Root biomass	1	280.381	80.7**	1	0.698	0.2ns	3	2.895	0.83ns
Stem biomass	1	168.330	79.51**	1	31.857	15.05**	3	57.630	27.22**
Leaf biomass	1	196.222	76.98**	1	18.612	7.30**	3	39.440	15.47**
Total biomass	1	1912.033	131.20**	1	83.225	5.71*	3	148.106	10.16**
Root volume	1	1321.425	458.03**	1	69.432	24.07**	3	0.0099	2.544*
Leaf area	1	1030.360	220.93**	1	47.262	10.13**	3	44.409	9.52**
Specific leaf area	1	24184.387	65.30**	1	2696.651	7.28**	3	2545.622	6.87*
Physiochemical									
Photosynthesis (A)	1	558.319	263.2**	1	1953.111	920.7**	3	27.563	12.99**
Transpiration (E)	1	82.19	263.79**	1	0.009	0.03ns	3	3.658	75.93**
Stomatal conductance (gs)	1	253891.5	46.35**	1	11172.8	2.04ns	3	91935.0	16.79**
temperature	1	370.84	11.42**	1	0.823	0.03ns	3	1282.9	39.52**
Mesophilic conductance (gm)	1	0.004	258.2**	1	0.016	1013.62**	3	0.0001	5.05*
Intracellular CO2 concentration (Ci)	1	8473.06	1.73ns	1	54885.59	11.18**	3	24535.26	5.00*
Relative water content (RWC)	1	1809.089	41.08**	1	636.563	14.45**	3	806.224	18.31**
Biochemical									
Chlorophyll a	1	0.554	33.23**	1	0.202	12.15**	3	0.001	0.10ns
Chlorophyll b	1	0.482	30.41**	1	0.518	32.73**	3	0.018	1.19ns
Chl a/b ratio	1	0.004	0.15ns	1	0.252	10.51**	3	0.113	4.69*
Total chl	1	2.070	36.04**	1	1.370	23.85**	3	0.009	0.16ns
Carotenoid	1	0.034	2.72ns	1	0.147	11.76**	3	0.002	0.17ns
Total chl/ Carotenoid	1	9.363	146.29**	1	0.289	4.52*	3	0.410	6.41*
Proline	1	0.003	0.04ns	1	0.203	2.61ns	3	0.610	7.83*
Malondialdehyde (MDA)	1	2.509	7.06*	1	0.897	0.17ns	3	0.739	0.17ns
Electrolyte leakage (EL)	1	101.036	46.35ns	1	584.924	6.06**	3	579.213	6.00*

ns, nonsignificant, *P < 0.05, **P < 0.01.

The height growth of both species was not significantly influenced by irrigation or the interaction between species and irrigation. Nonetheless, species identity had a significant impact on height growth with oak SH surpassing that of wild pear seedlings by 76.6%, irrespective of the irrigation treatment (**Figure 2B**). Root biomass in both species remained unaffected by drought and overall, the root biomass of oak seedlings was 2–3 times higher than that of wild pear seedlings (**Table 2**, **Figure 2C**). Under water deficit conditions, a significant reduction in stem biomass was only observed in oak (-22.6%), resulting in a significant species × drought interaction (**Table 2**, **Figure 2D**). However, oak seedlings had significantly greater biomass than pear to start with. Leaf biomass declined by 13.8% in wild pear and by 32.5% in oak in response to drought conditions, which also gave rise to a significant species × drought interaction (**Table 2**, **Figure 2E**). The pattern seen in above-ground biomass compartments was also evident in the total biomass with stronger losses in oak (-25%) relative to pear (-14%), reflected in a significant species × drought interaction (**Table 2**, **Figure 2F**). Oak root volume was roughly 4.7 times larger than that of wild pear. Furthermore, in both irrigation regimes, oak seedlings consistently exhibited a greater root volume compared to wild pear seedlings (3 times and 2.4 times at FC100 and FC40, respectively) (**Figure 2G**). Both total leaf area and SLA remained largely unaffected by drought in pear but showed steep declines in oak (leaf area: - 22%, SLA: -28%) resulting in a significant species x drought interaction (**Table 2**, **Figures 2H, I**). Overall, at each soil field capacity, the SLA was significantly greater in oak than in wild pear (**Figure 2I**).

### 3.2 Physiological characteristics

**Figure 3:**
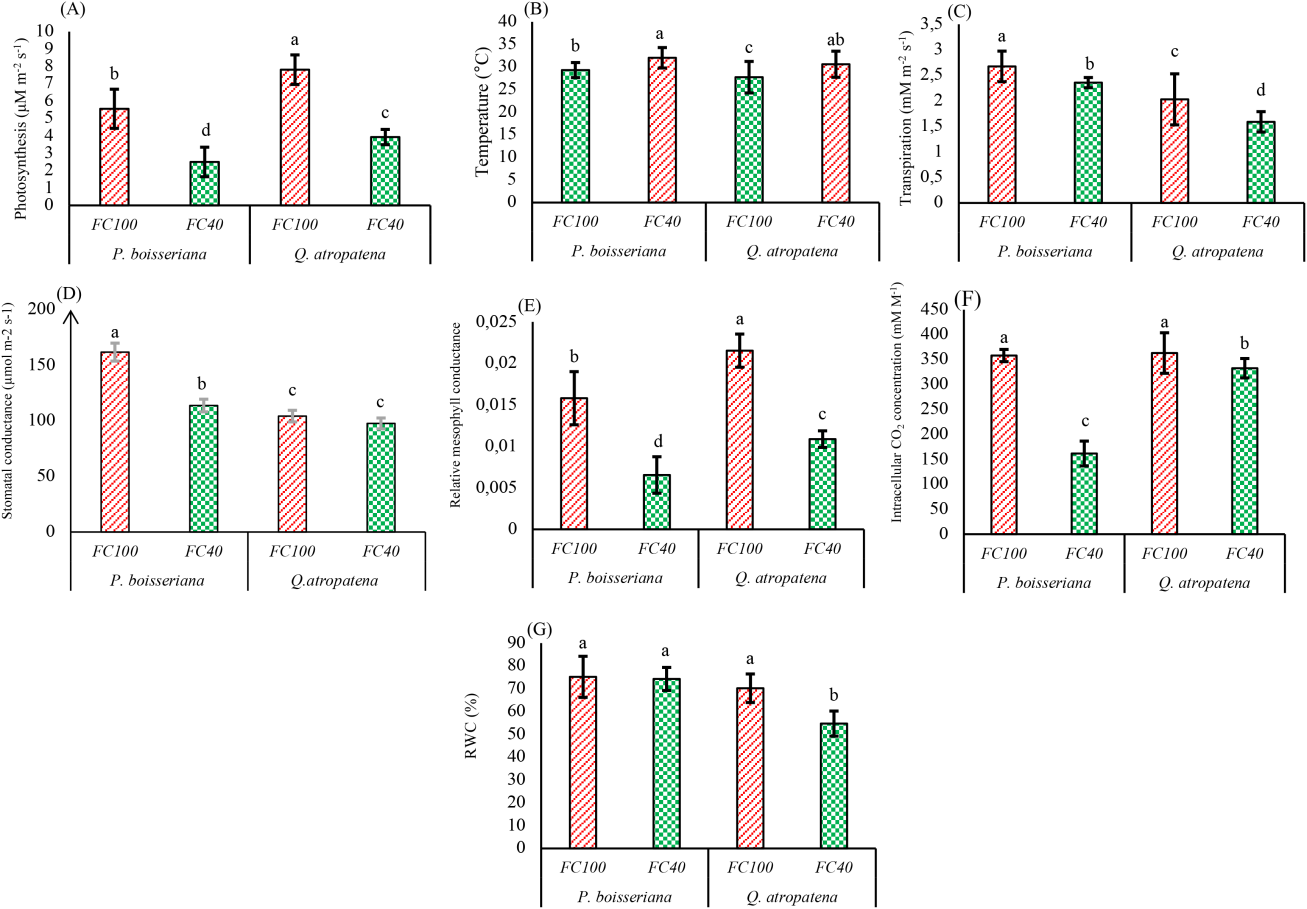
Means of **(A)** photosynthesis, **(B)** temperature, **(C)** transpiration, **(D)** stomatal conductance, **(E)** intracellular CO_2_ concentration, **(F)** relative mesophyll conductance and **(G)** RWC of *Quercus atropatena* and *Pyrus boisseriana* seedlings under well-watered and water deficit conditions. Different lowercase letters indicate significant differences between means among the 4 treatment combinations (α = 0.05). Different uppercase letters indicate significant differences between the two tree species, regardless of the irrigation treatment (α = 0.05).

The results of the two-way analysis of variance demonstrated that the interaction of water deficit stress and species type had a significant effect on all physiological characteristics of oak and wild pear seedlings (**Table 2**). Under water deficit stress, *A* rates in wild pear and oak diminished by 55% and 49.6%, respectively, while oak consistently exhibited higher photosynthetic carbon uptake than wild pear across both water conditions (**Figure 3A**). Additionally, leaf temperature increased under water deficit stress in both species (**Figure 3B**). Furthermore, drought conditions led to a reduction in E rates in both species, with wild pear experiencing an 11.8% decrease and oak a 21.7% decrease (**Figure 3C**). The g_s_ did not change significantly under drought conditions in oak seedlings, while drought-exposed pear seedlings showed a significant reduction by 19.7% (**Figure 3D**).

The g_m_-rel was generally greater in oak compared to pear but declined under drought in both species, with wild pear experiencing a 58.5% decrease and oak a 49.5% decrease (**Figure 3E**). In the FC40 treatment, oak exhibited higher *C*_i_ levels than wild pear. The drought-related decrease in *C*_i_ in wild pear was significantly more pronounced (-54.8%) compared to oak, which only showed a slight decrease of -8% under stress conditions (**Figure 3F**). The RWC remained unaffected by drought conditions in wild pear but decreased by 22% in oak (**Figure 3G**).

### 3.3 Biochemical characteristics

**Figure 4:**
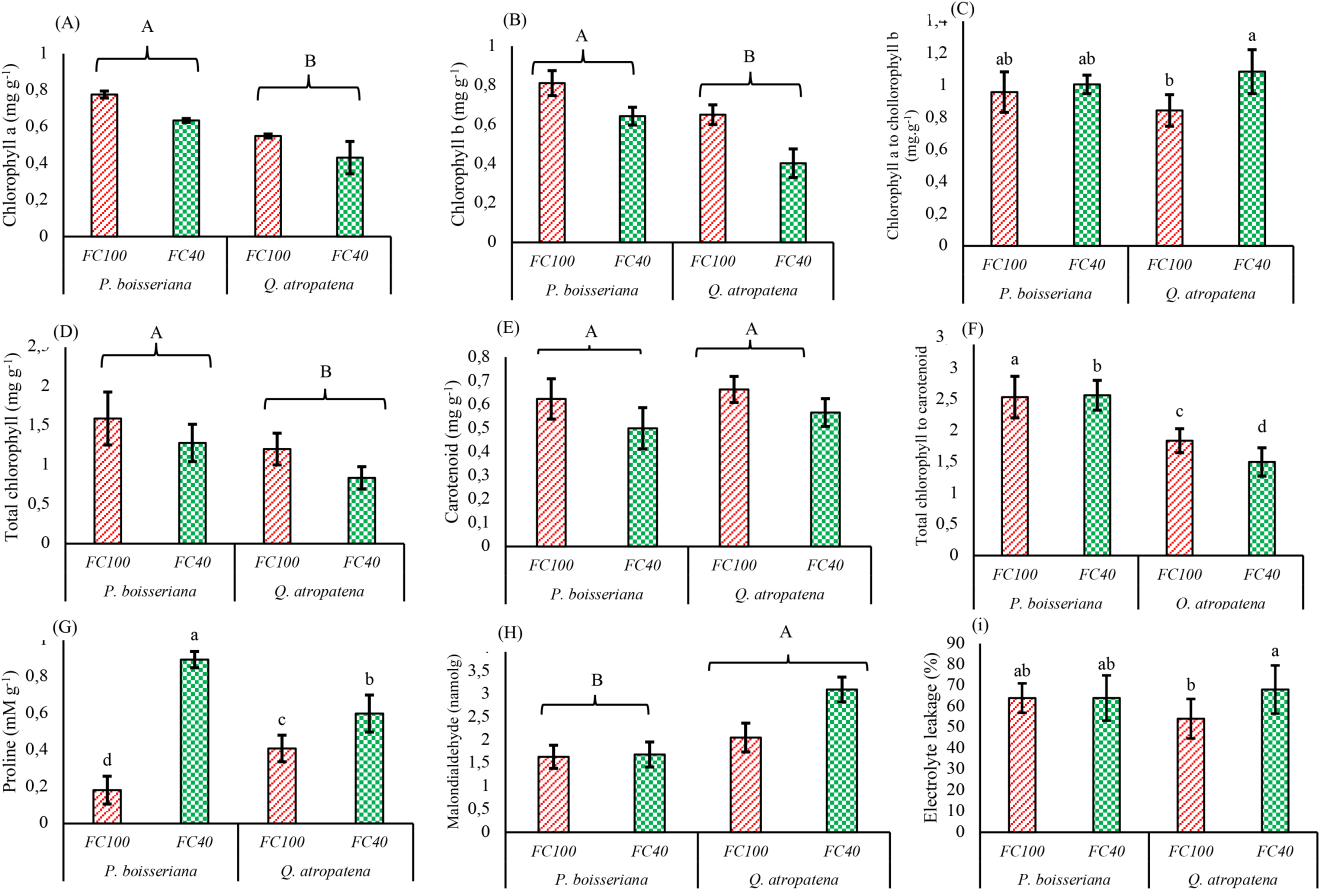
Means of **(A)** chlorophyll a, **(B)** chlorophyll b, **(C)** chlorophyll a / chlorophyll b ratio,**(D)** total chlorophyll content, **(E)** carotenoid content, **(F)** total chlorophyll/total carotenoidratio, **(G)** proline concentration, **(H)** malondialdehyde concentration, and (I) electrolyte leakageof Quercus atropatena and Pyrus boisseriana seedlings under well-watered and water deficitconditions. Different lowercase letters indicate significant differences between means amongthe 4 treatment combinations (α = 0.05). Different uppercase letters indicate significantdifferences between the two tree species, regardless of the irrigation treatment (α = 0.05).

There were no significant species × drought interactions on chlorophyll a or b, total chlorophyll, carotenoids or MDA (**Table 2**). Nonetheless all these parameters were significantly affected by the two main effects, except for carotenoids which were similar among species but declined significantly under water deficit (wild pear: -19.9%, oak: -14.5%). However, MDA levels in oak were significantly greater by about 32% compared to wild pear but remained unaffected by the water deficit (**Table 2**, **Figure 4**). Regardless of species identity, the amounts of chlorophyll a, chlorophyll b, and total chlorophyll were 30.4%, 27.5%, and 28.9% higher in wild pear than in oak, respectively (**Figures 4A, B, D**). We detected significant species × drought interactions on the chlorophyll a/b ratio, the chlorophyll/carotenoid ratio, proline content and EL (**Table 2**, **Figure 4**). More specifically, the chlorophyll a/b ratio remained largely unaffected by water deficit in wild pear but increased by ca. 24% under water deficit in oak seedlings (**Figure 4C**). The chlorophyll/carotenoid ratio did not change significantly under water deficit in wild pear but decreased by ca. 18% in oak (**Figure 4F**). Under water deficit, proline levels rose about 4.9-fold in wild pear but only 32% in oak (**Figure 4G**). Electrolyte leakage was unresponsive to water deficit in wild pear but increased by 20.4% in FC40-exposed oak seedlings (**Figure 4I**).

**Figure 5:**
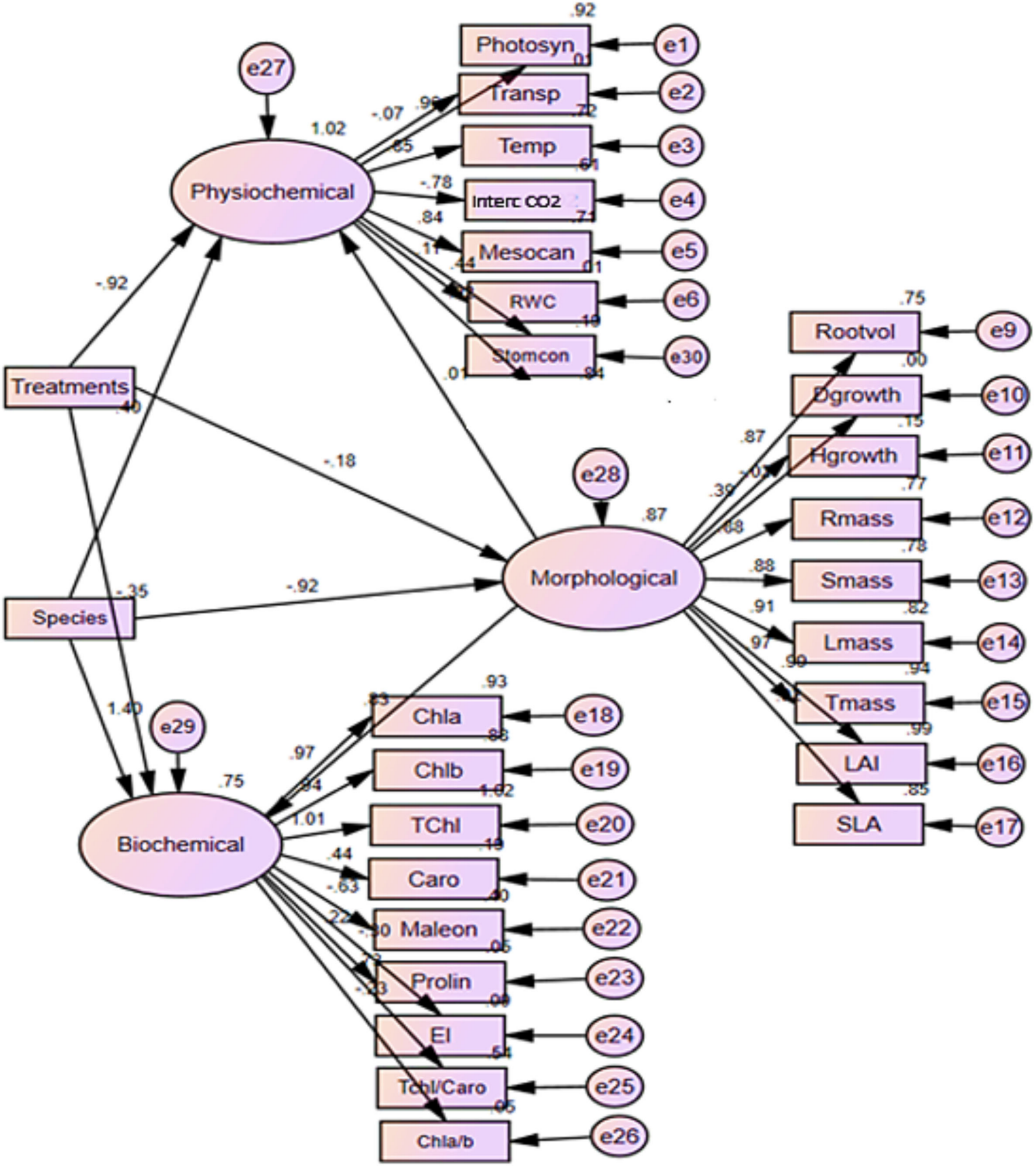
Structural equation model (SEM) evaluating the direct and indirect effects of drought stress on some morphological, physiological, and biochemical traits in oak (*Quercus atropatena* Schwarz.) and wild pear (*Pyrus boisseriana* Buhse) seedlings. The numbers on the rectangular boxes and directions represent the standardized variance and the standardized regression weights, respectively. Abbreviations are detailed in the parentheses (Rootvol, Root volume; Dgrowth, Diameter growth; Hgrowth, Height growth; Rmass, Root biomass; Smass, Stem biomass; Lmass, Leaf biomass; Tmass, Total biomass; LAI, Leaf Area; SLA, Specific leaf area; Photosyn, Photosynthesis; Transp, transpiration; Stomcon, Stomata conductance; Temp, temperature; IntercCO_2_, Intercellular CO_2_ concentration; Mesocan, Relative mesophyll conductance; RWC, Relative water content; Chla, Chlorophyll a; Chlb, Chlorophyll b; Chla/b, Chlorophyll a / Chlorophyll b; Tchl, Total chlorophyll; Caro, Carotenoid; TchlCaro, Total chlorophyll/ Carotenoid; Malon, Malondialdehyde; El, Electrolyte leakage).

**Figure 6:**
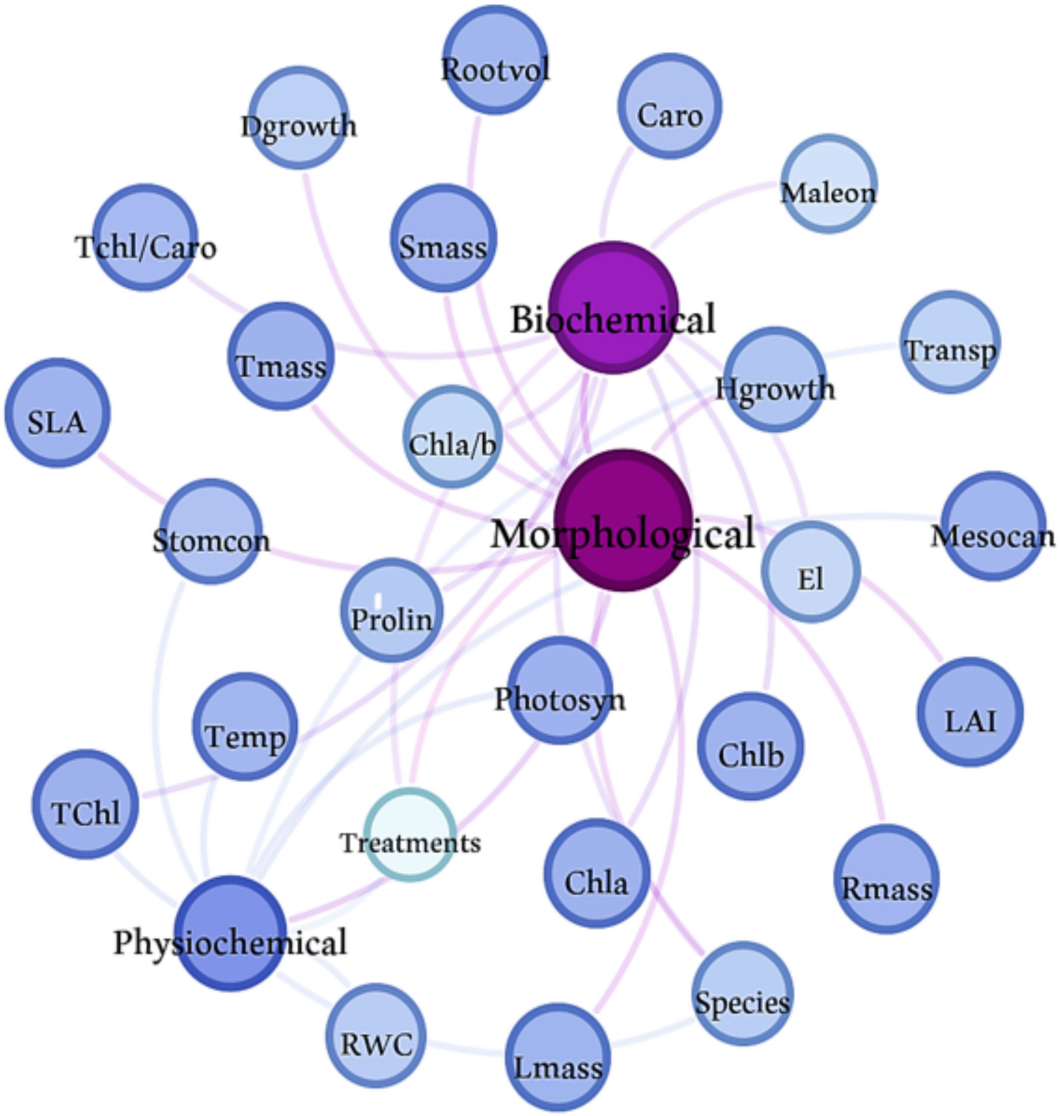
A network model generated using drought stress in oak (*Quercus atropatena* Schwarz.) and wild pear *(Pyrus boisseriana* Buhse) seedlings and 24 morphological, physiological, and biochemical traits. Each node (circle) represents a property, and each edge (connection) indicates significant correlations among properties and samples. Node colors reflect the identified modules (groups), and the size and label of each node is proportional to the eigenvector centrality. The thickness of each edge (line) between two nodes is proportional to the standardized regression weights. The size and color density of the lines reflects the varying strength of relationship between the 24 morphological, physiological, and biochemical traits, i.e., thicker lines indicate stronger relationships and the direction of the arrows shows the influence of the factors on each other. Some variables are more central and have more connections than others. Abbreviations are detailed as follow.

The structural equation model indicated that both species identity and water deficit stress significantly influenced the variations in physiological and biochemical parameters by affecting morphological traits. These traits were carefully chosen and incorporated into the SEM (**Figure 5**). The results from the network model reveal that the roles of species identity and water deficit stress substantially impact the traits under investigation (**Figure 6**). Notably, species identity exerts a more pronounced influence on biochemical traits, while water deficit stress primarily affects physiological and morphological traits.

## 4 Discussion

### 4.1 Morphological characteristics

This research examined morphological, physiological and biochemical drought responses of seedlings of two prevalent tree species from the Hyrcanian forests to provide guidance to stakeholders concerned with nursery propagation and ecosystem management. Generally, the ability to survive periods of water scarcity and minimize growth losses associated with water deficit stress is essential for understanding plant drought tolerance (Engelbrecht and Kursar, 2003). In our study, the average survival rates across treatments exceeded 90% in both species, which, along with the relatively small losses in height and diameter growth under drought, indicates a remarkable drought tolerance. Numerous studies have shown that water scarcity often has a negative impact on plant survival rates. For instance, drought experiments conducted on *Robinia pseudoacacia* seedlings by Norouzi Haroni and Tabari (2015) and on pine seedlings by Guo et al. (2010) showed that elevated drought stress caused reduced soil water availability and resulted in about 20% lower survival rates in these plants.

Under drought conditions, plants typically prioritize resource allocation to belowground sinks, i.e. increasing root volume and length to improve water uptake, thereby enhancing drought tolerance (Rooki et al., 2018). Indeed, in our study root volume increased in both species under drought but since this occurred without concomitant increases in root biomass, we can confidently conclude that root expansion was facilitated by a reduction in root density. Typically, the earliest morphological indicators of drought stress include decreases in SH growth, stem diameter growth, and total biomass production, since growth processes become increasingly impaired as drought conditions intensify (Monclus et al., 2006). Reduced growth during drought stress is primarily linked to a decrease in turgor pressure in the cambium and inadequate cell development (Peters et al., 2021; Ali et al., 2023), ultimately leading to a reduction in shoot growth, in diameter and/or height. In our study, significant drought-induced radial growth losses only occurred in wild pear while height growth showed no significant reductions in either species. The observed decrease in diameter growth of wild pear by 38% is consistent with the results reported for *Cupressus arizonica* and *Cupressus sempervirens* var. *fastigiata* (Rooki et al., 2018), *Pinus nigra* (Dehghan et al., 2016), and *Cercis siliquastrum* (Norouzi Haroni et al., 2017). The smaller diameter growth in drought-stressed wild pear seedlings was not accompanied by a reduction in stem biomass. Together with the small but statistically insignificant reduction in height growth, this suggests an increase in wood density, as reported previously for other woody species growing under water deficit (Lambers et al., 2008; Eliyani and Koesmaryono, 2005). Our findings showed drought stress-induced reductions in stem biomass (oak: -22.6%), leaf biomass (oak: -32.5%, wild pear: -13.8%) and total biomass (oak: -30.2%, wild pear: -12.2%) of seedlings. These results are consistent with the study of Jafarnia et al. (2018) on seedlings of oak species (Q*. libani*) with two different seed provenances showing a 15-35% reduction in total dry biomass, Dehmardy et al. (2018) on olive seedlings (*Olea europaea*), Ashkavand et al. (2016) on hawthorn seedlings (*Crataegus aronia* L.) and mahaleb cherry (*Prunus mahaleb* L.), and Saeidi Abueshaghi et al. (2023) on Judas seedlings (*Cercis siliquastrum* L.) with a ca. 46%, and 45% reduction in total fresh and dry biomass, respectively. As outlined above, the reduction in plant shoot biomass observed during drought conditions is probably primarily due to a decline in turgor pressure, which adversely affects the growth and proliferation of plant cells (Ashkavand et al., 2016). Another contributing factor is the decrease in E levels, which plants reduce to manage drought stress, ultimately leading to less shoot growth (Yang et al., 2007). During periods of drought stress, oak and wild pear seedlings undergo specific physiological changes, which help them survive in their environment. This starts with a tighter stomatal control over E. When water is scarce, the seedlings retain more of the water taken up by the roots within the root system to promote stronger root growth or root volume. In other words, during periods of water deficit, plants tend to extend their roots deeper into the deeper soil layers to tap into soil moisture reserves and thus mitigate drought stress (Norouzi Haroni et al., 2017), which contributes to an increase in root volume and root weight. In our study, the root volume increased significantly (33% for wild pear and 13% for oak), although this was not associated with significant changes in root weight under drought conditions, translating into a decrease in root tissue density. Similarly, a study involving seedlings of *Pistacia lentiscus* L. and *Quercus coccifera* found that heightened drought conditions do not necessarily promote the development of plant root systems, i.e. greater root biomass (Vilagrosa et al., 2003).

In the present study, under drought conditions, leaf area and SLA in oak decreased by 22% and 28%, respectively, which is consistent with the findings reported for *Cercis siliquastrum* (Norouzi Haroni et al., 2017), *Pistacia atlantica* (Mirzaei and Karamshahi, 2015), *Celtis Caucasica* (Sepahvand et al., 2021), *Populus euphratica* (Zhao et al., 2021), and *Ligustrum obtusifolium* (Wang et al., 2024a). Leaf area is primarily determined by leaf tissue turgor, temperature, and various growth factors, which tend to decline under conditions of water deficit stress (Wang et al., 2020). This leads to a reduction in leaf area, primarily due to decreased E, cell division, and the elongation of cells during periods of water scarcity (Yang et al., 2025). Generally, based on various morphological traits our findings indicated that oak and wild pear seedlings exhibit significant drought stress tolerance. Taken together, our results imply that, similar to other oak and pear species, the high drought tolerance of *Q. atropatena* and *Pyrus boissieriana* appears to be primarily attributed to the ability to develop an extensive and deep root system allowing these species to tap into deeper-lying water and nutrient pools (Abrams, 1990; Thomas and Gausling, 2000; Zarafshar et al., 2014b).

### 4.2 Physiological characteristics

The present study indicated that *A* attained greater rates in oak seedlings compared to wild pear both under high and low water availability. In line with previous findings, the rate of *A* declined with decreasing water deficit (from FC100 to FC40) (Jafarnia et al., 2018; Xiong et al., 2022). The g_s_ in our oak seedlings remained unchanged by water scarcity, which has also been reported by Thomas and Gausling (2000) for seedlings of two European oak species (*Q. robur, Q. petraea*) and the same water use behaviour has recently been reported for mature individuals of the same oak species (Bader et al., 2022). This response differs from the pattern typically seen under water deficit, which is characterised by declining leaf water potential, followed by reductions in stomatal aperture and hence lower g_s_. Decreases in g_s_ result in reduced gas-exchange, thereby impairing the rate of *A* and ultimately hindering growth (Yang et al., 2007, 2021). In the current study, E decreased with increasing water deficit, which is consistent with the results of Mirzaei and Karamshahi (2015) on *Pistacia atlantica* seedlings and Rooki et al. (2018) on *Cupressus arizonica* and *Cupressus sempervirens*. The decline in E rates observed in stressed wild pear seedlings is likely due to stomatal downregulation. Such a stomatal restriction of E is likely co-regulated by hydraulic cues at the leaf-level and long-distance signaling from the roots via the abscisic acid pathway. Reduced E rates during periods of drought stress are an effective strategy to preserve a favorable leaf water status, thereby helping to avert plant mortality under such conditions (Fang and Xiong, 2015). By contrast, the diminished E in oak under water deficit must be due to non-stomatal limitations (e.g. xylem embolism) since g_s_ remained unchanged. It should be emphasized though, that the oak seedlings in our study showed consistently lower g_s_ and E and hence more economical water use than wild pear. Such conservative water relations have also been reported for mature oak species in Central Europe (Bader et al., 2022).

Leaf surface temperature in both species increased with decreasing soil water availability, which is consistent with the findings of Mirzaei and Karamshahi (2015) and Rooki et al. (2018) and reflects the reduced transpiration cooling associated with non-stomatal downregulation of E (Scherrer et al., 2011). Under drought conditions, g_s_ exhibited a decline, a finding corroborated by previous research (Hosseinian et al., 2018). Our research also showed a significant decrease in *C*_i_ during water deficit conditions for both species, with a greater reduction in wild pear compared to oak. In wild pear the large drop in *C*_i_ seems more closely linked to the observed decrease in g_s_, resulting in diminished CO_2_ absorption. In oak, the rather high *C*_i_, coinciding with unaltered g_s_, may result from increased photorespiration and, as a direct consequence, an accumulation of CO_2_ in the mesophyll. This sequence of events disrupts leaf gas-exchange processes and lowers the water use efficiency of the plant (Anjum et al., 2011). Reducing the soil water availability from field capacity to 40% thereof, caused a decrease in leaf RWC of oak, while the unaltered leaf RWC in wild pear suggests a more effective maintenance of leaf hydration status through tighter stomatal control and probably osmotic adjustment as indicated by the strongly elevated levels of proline, which, among other functions, also serves as an osmoregulant (see below). The RWC response to drought seen in oak, is consistent with the results of Dehghan et al. (2016) on *Pinus nigra* seedlings, and with those of Ashkavand et al. (2016) on *Crataegus aronia* seedlings. Leaf RWC is a very good indicator of the water status of the plant cell (Schonfeld et al., 1988). During periods of drought, reduced moisture within the leaves leads to reduced cell expansion, which subsequently reduces leaf biomass.

### 4.3 Biochemical characteristics

Our findings indicate notable differences in the biochemical characteristics examined under conditions of water deficit stress, with the exception of carotenoids and MDA. A degradation of photosynthetic pigments due to drought stress is a typical response observed in numerous plant species (Farooq et al., 2012). Most plants exhibit a decline in chlorophyll content as drought stress intensifies. Similar to the present study, Liu et al. (2022), reported a decrease in chlorophyll content under drought stress conditions in three *Juglans* species. Variations in chlorophyll content are often associated with the plant’s water availability (Gitelson et al., 2003). Consistent with the results from *Q. infectoria and Q. libani* (Ghanbary et al., 2021) and *Olea europaea* (Dehmardy et al., 2018), in the present study, a decrease in carotenoid content was observed under drought conditions. Drought stress also impairs photosynthetic performance by degrading photosynthetic pigments, leading to a decline in chlorophyll and carotenoid content (Tatari et al., 2020). This reduction may result from inhibited or halted synthesis of photosynthetic pigments under water-deficit conditions (Wang et al., 2021). In addition, ROS accumulating under drought stress can directly oxidize pigments or cause oxidative damage to cell membranes, thus affecting the structural integrity of chloroplasts and pigment-binding proteins.

In the present study, proline accumulated in both species under drought stress conditions, but the increase was greater in wild pear than in oak. This drought-related increase in proline is in line with the responses observed in other tree species (*Pinus eldarica*: Delafan Azari et al., 2018; *Cercis siliquastrum*: Saeidi Abueshaghi et al., 2023; *Lagerstroemia indica*: Wang et al., 2021), especially in *Quercus brantii* (Jafarnia et al., 2018) and *Q. infectoria* (Ghanbary et al., 2020), which are native to the Zagros forest region in Iran. However, no increase in proline was reported under water deficit for the more drought-sensitive *Quercus libani*, which co-occurs in the Zagros forests (Ghanbary et al., 2020). The extent of proline accumulation in plant cells depends on the type of species and the severity of stress (Claussen, 2005). The observed rise in proline content could result from either enhanced synthesis (increased anabolism) or reduced breakdown (decreased catabolism) of proline. In the case of Persian oak (*Quercus brantii*), severe drought conditions resulted in an increase in proline and MDA levels, as well as elevated EL (Jafarnia et al., 2018). Although in the present study, MDA levels did not change significantly with irrigation regime, oak showed a greater content than pear. By contrast, the two native oaks from the Zagros forests, *Q. infectoria* and *Q. libani*, both showed a substantial increase in MDA in response to drought (Ghanbary et al., 2020). Similar findings were reported by Wang et al. (2021) for *Lagerstroemia indica*, Lim et al. (2017) for *Quercus acutissima*, and Saeidi Abueshaghi et al. (2023) for *Cercis siliquastrum*, indicating that the amount of MDA increases with the intensity of drought in many woody species. Indeed, most plants exhibit a common response to oxidative stress in their biological membranes through lipid membrane peroxidation, which is indicated by a rise in MDA (Jafarnia et al., 2018). Specifically, during periods of drought stress, the overproduction of ROS leads to oxidative damage, potentially culminating in plant death. This finding reinforces the notion that MDA content is directly linked to the stress sensitivity of various plant species (Zhang et al., 2020).

In our study, EL was markedly raised in our oak seedlings under drought, which is consistent with the findings for Persian oak (*Quercus brantii*; Jafarnia et al., 2018), Aleppo oak (*Q. infectoria*) and Lebanon oak (*Q. libani*) (Ghanbary et al., 2021), the three prevailing oak species in the Zagros forests, the other major forest zone of Iran. Under drought conditions, the plasma membrane of plant cells is one of the first structures to become damaged, which increases EL through the loss of the selective permeability property of the cell (Farooq et al., 2012) and is accompanied by an increase in the production and accumulation of ROS (such as superoxide radicals, hydrogen peroxide, and hydroxyl radicals) (Wang et al., 2024a). An increase in lipid peroxidation, together with a reduction in the membrane stability index, has been widely recognized as a major factor contributing to elevated EL under drought stress (Fayyaz et al., 2013).

## 5 Conclusion

Although both *Pyrus boissieriana* and *Quercus atropatena* are generally considered drought-tolerant species, our findings show clear differences in their drought responses. Oak exhibited larger reductions in stem, leaf, and total biomass as well as decreases in SLA and leaf area, indicating a stronger morphological sensitivity to water deficit. In contrast, wild pear showed smaller biomass losses and stronger accumulation of proline, reflecting more effective osmotic adjustment. These patterns suggest that, while both species fit within the drought-tolerant category, wild pear displayed relatively greater tolerance than oak in morphological and biochemical terms. Severe drought conditions caused physiological impairment in both species (e.g., reductions in *A*, intracellular CO_2_ concentration, g_s_ and RWC of leaves), but oak maintained greater *A* rates and showed more conservative water use under water deficit. Because selecting appropriate tree species is essential for maintaining long-term forest sustainability, our results offer important insights into the species-specific tolerance and adaptive responses to drought during the vulnerable seedling stage. Our study provides valuable information for nursery managers dealing with water scarcity and for those involved in afforestation initiatives within the arid and semi-arid regions of the Hyrcanian forests, to guide best practices in seedling production and planting for these two species. The suitability of *Pyrus boissieriana* and *Quercus atropatena* for afforestation depends on site-specific objectives, with each species offering distinct advantages under drought-prone conditions.

## Data Availability

The raw data supporting the conclusions of this article will be made available by the authors, without undue reservation.
